# Assessment of Combined Karyotype Analysis and Chromosome Microarray Analysis in Prenatal Diagnosis: A Cohort Study of 3710 Pregnancies

**DOI:** 10.1155/2022/6791439

**Published:** 2022-12-29

**Authors:** Jin Wang, Danni Wang, Yan Yin, Yi Deng, Mengling Ye, Ping Wei, Zhuo Zhang, Chun Chen, Shengfang Qin, Xueyan Wang

**Affiliations:** Department of Medical Genetics and Prenatal Diagnosis, Sichuan Provincial Maternity and Child Health Care Hospital, Chengdu 610003, China

## Abstract

**Objective:**

The current study aimed to compare the characteristics of chromosome abnormalities detected by conventional G-banding karyotyping, chromosome microarray analysis (CMA), or fluorescence in situ hybridization (FISH)/CNVplex analysis and further explore the application value of combined karyotype analysis and CMA in prenatal diagnosis with a larger sample size.

**Methods:**

From March 2019 to March 2021, 3710 amniocentesis samples were retrospectively collected from women who accepted prenatal diagnosis at 16 to 22 + 6 weeks of pregnancy. The pregnant women underwent karyotype analysis and CMA. In the case of fetal chromosomal mosaicism, FISH or CNVplex analysis was utilized for validation.

**Results:**

In total, 3710 G-banding karyotype results and CMA results from invasive prenatal diagnosis were collected. Of these, 201 (5.41%) fetuses with an abnormal karyotype were observed. The CMA analysis showed that the abnormality rate was 9.14% (340/3710). The detection rate of CMA combined with karyotype analysis was 0.35% higher than that of CMA alone and 4.08% higher than that of karyotyping alone. Additionally, 12 cases had abnormal karyotype analysis, despite normal CMA results. To further detect the chromosome mosaicism, we used FISH analysis to correct the karyotype results of case 1. Correspondingly, a total of 157 cases showed abnormal CMA results but normal karyotype analysis. We also found chromosomal mosaicism in 4 cases using CMA. Moreover, CNVplex and CMA demonstrated that representative case 15 was mosaicism for trisomy 2.

**Conclusions:**

Conventional G-banding karyotyping and CMA have their own advantages and limitations. A combination of karyotype analysis and CMA can increase the detection rate of chromosome abnormalities and make up for the limitation of signal detection.

## 1. Introduction

A birth defect is a developmental abnormality that occurs during pregnancy. When an ultrasound examination indicates anomalies, further diagnosis using chromosome analysis will be conducted [1]. Since the mid-1960's, G-band chromosomal karyotyping has been widely used for testing genetic abnormalities in clinical practice because it shows clear chromosome bands, can be distinguished under an ordinary microscope, the specimen can be stored for a long time, etc. However, this approach has several limitations, such as a long detection period and low resolution [2]. With the improvement of diagnostic methods, chromosome microarray analysis (CMA) is emerging to identify small microdeletions and duplications, which is helpful to understand disease-related copy number variations (CNVs) [3]. Additionally, previous studies have further demonstrated that CMA is superior to conventional karyotyping analysis [4] and can be deemed a first-line test in pregnancies with a priori low risk [5]. In clinical settings, the combination of conventional karyotype analysis and CMA is often used for prenatal diagnosis.

Based on the clinical use of two methods, a previous study demonstrated that differences between the two methods may lead to different results in the diagnosis of aneuploid chromosomes [6]. According to statistics, approximately 32% of fetuses with structural abnormalities have a clinically relevant abnormal karyotype, and an additional 6% have causative CNVs [7]. Therefore, it is necessary to evaluate more comprehensively the difference between karyotyping analysis and CMA and the combination of these two methods in the diagnosis of fetuses with structural abnormalities. Here, we explored the characteristics of chromosome abnormalities detected by conventional G-banding karyotyping and CMA in fetus cases and further compared the detecting results in a larger sample size using different testing techniques. Additionally, fluorescence in situ hybridization (FISH) analysis or CNVplex analysis was conducted to evaluate chromosome mosaicism. Those findings showed that the combination of conventional karyotyping analysis and CMA identified the additional, clinically significant cytogenetic information, indicating the value of the combination of the two methods.

## 2. Materials and Methods

### 2.1. Patients

In total, 3710 amniocentesis samples were retrospectively collected from women at 16 to 22^+6^ weeks of pregnancy in our hospital. All pregnant women underwent karyotype analysis and CMA. The inclusion criteria were as follows: (1) high-risk results from serological screening; (2) high-risk results from noninvasive DNA prenatal screening, including trisomy 21, 13, 18, and other chromosome abnormalities; (3) advanced age (over 35 years); (4) high-risk results from ultrasound genetic marker screening (NT > 95th, nasal bone dysplasia, mild expansion of the lateral ventricle); (5) fetus abnormalities, including fetal intrauterine growth restriction and abnormal fetal structure. Written consent was obtained from each pregnant woman. The study was approved by the Ethics Committee of our hospital.

### 2.2. Karyotype Analysis

Amniotic fluid (20 mL) was collected from pregnant women at 16 to 22^+6^ weeks. Samples were cultured according to standard cytogenetic protocols [8]. Giemsa-banding staining was utilized to analyze the cultured amniocytes, and then karyotype analysis was conducted.

### 2.3. CMA

DNA from amniotic fluid cells was extracted by the Puregene Cell and Tissue Kit (QIAGEN, Beijing, China). Following, the Goldeneye DNA ID System 20A (Peoplespot, Beijing, China) was used for maternal cell contamination (MCC) identification. For CMA testing in MCC-free samples, a well-established customized 180 k CGX™ SNP v1.1 chip (Agilent Technologies, Santa Clara, CA, USA) was employed. The data were analyzed using Genoglyphix software (PerkinElmer, Waltham, MA, USA).

### 2.4. Fluorescence in Situ Hybridization (FISH) Analysis of Chromosome Mosaicism

An amniotic fluid sample was centrifuged for 10 min to obtain the cell precipitate. CSP18/CSPX/CSPY probes (Jinpujia Company, Beijing, China) were used for FISH detection of cultured amniotic fluid cells [9]. The fluorescence signal was detected and then calculated the chimeric ratio. At the same time, fluorescence image acquisition of metaphase cells with abnormal signals was carried out.

### 2.5. CNVplex Analysis of Chromosome Mosaicism

CNVplex, a high-throughput analysis (Genesky, Shanghai, China), mainly includes hybridization, ligation, and multiplex PCR amplification. The product was amplified by AB 3500Dx Analyzer capillary electrophoresis, and the data was analyzed by GeneMapper software, referring to the manufacturer's instructions.

## 3. Results

**Figure 1:**
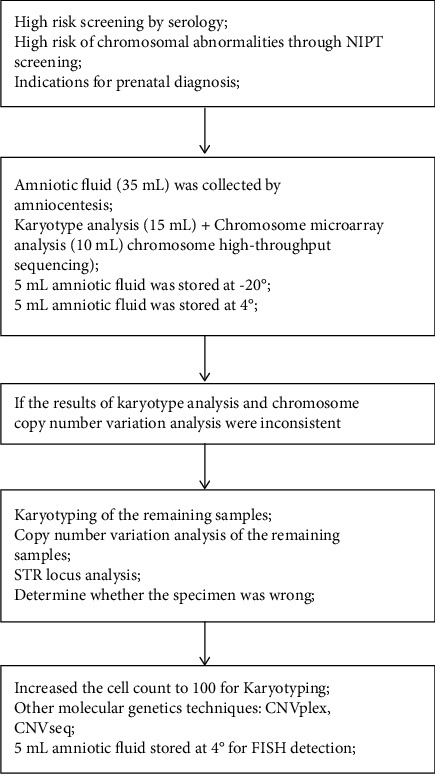
The flow chart of the disease diagnosis.

In total, 3710 pregnant women underwent amniocentesis to obtain amniotic fluid for karyotype analysis, and CMA was collected. The flow chart for the disease diagnosis is shown in Figure 1. Of these, 201 (5.41%) pregnant women with fetal karyotype abnormal results were detected. CMA analysis showed that the abnormality rate was 9.14% (340/3710), and CNVs and loss of heterozygosity (>10 Mb) accounted for 339 cases (9.13%). Additionally, karyotype analysis and CMA analysis abnormalities were found in 352 pregnant women (9.49%) (Table 1).

### 3.1. Comparison of Karyotype Results in Abnormality Group and CMA Results in Normality Group

**Figure 2:**
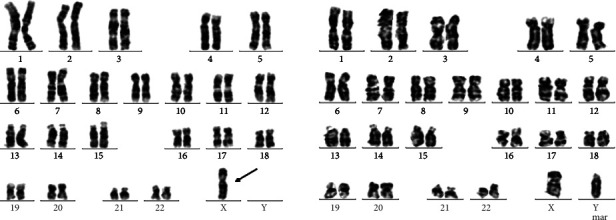
The results of G-banding karyotype analysis in case 1 showed the 45, X, [73]/46, X, +mar [10].

**Figure 3:**
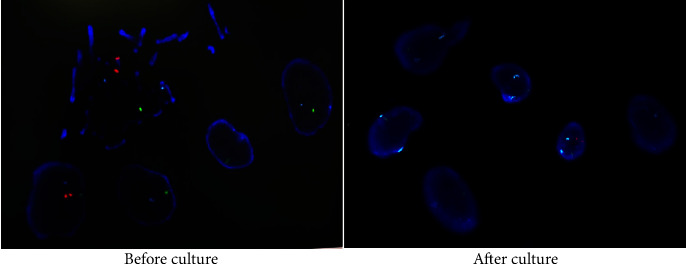
FISH analysis in case 1 was further used to evaluate the chromosome abnormalities.

As shown in Table 2, 12 cases had abnormal karyotype analyses but normal CMA results. Among them, 6 cases involved two or more chromosomal translocations, and 5 cases had a low proportion of mosaicism. Especially, 1 case was 45, X, [73]/46, X, +mar [10] (Figure 2). After the elimination of sample errors and maternal contamination, FISH indicated that the mar chromosome was the fusion of two Y chromosomes, similar to the dose of 47, XYY. Before culture, FISH results depicted that the signals of X and XYY were approximately equal, indicating the normal male; however, after culture, the signals of X and XYY were 71% and 29%, indicating that 45, X cells grew with selective dominance (Figure 3). Thus, combined with FISH results, karyotype results were modified to 45, X, [73]/46, X, psu dic(Y) (q12) [10].

### 3.2. Comparison of Karyotype Results in the Normality Group and CMA Results in the Abnormality Group

**Figure 4:**
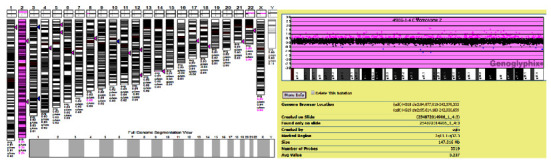
CMA results in case 15 indicated mosaicism for trisomy 2.

**Figure 5:**
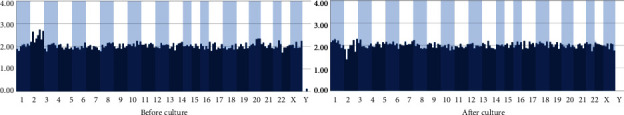
Comparison of CNVplex before and after culture in case 15.

A total of 157 cases showed abnormal CMA results but normal karyotype analysis. Additionally, we also found that 4 cases were chromosomal mosaicism (Table 3). In brief, the representative CAM results of case 15 represented the mosaic duplication of chromosomes (about 23.7%), indicating mosaicism for trisomy 2 (Figure 4). Correspondingly, CNVplex results showed that the signal of chromosome 2 in case 15 was between 2 and 3 before culture, suggesting mosaic trisomy 2. After culture, the signal of chromosome 2 was approximately equal to 2, suggesting that mosaic trisomy 2 was selective with no growth advantage *in vitro* (Figure 5).

## 4. Discussion

In China, a growing number of pregnant women are willing to accept additional molecular technology for prenatal diagnosis based on conventional karyotype analysis. Correspondingly, Shi et al. [10] have investigated the methods of prenatal diagnosis in the multicenter center and demonstrated that combined karyotype analysis and CMA are commonly used to assess pregnant women with other abnormal indications. Here, we found that conventional karyotype analysis and CMA can identify the cytogenetic differently information, which indicated the significant value of the combination of two methods for prenatal diagnosis.

According to statistics, approximately 6% of fetuses have chromosome abnormalities detected during amniocenteses, and the combined risk of a serious congenital anomaly in a fetus with translocations and inversions was estimated at 6.7% [11]. In the current study, karyotype abnormal results were detected in 201 (5.41%) of 3710 fetuses, which was similar to the proportion reported previously. Karyotype analysis is the gold standard for detecting chromosome abnormalities, but it is obviously inadequate for diagnosing chromosome mosaicism and estimating chromosome recombination. In 2013, the American College of Obstetrics and Gynecology (ACOG) recommended using CMA instead of traditional karyotype analysis when the fetus has one or more ultrasound abnormalities and requires invasive prenatal diagnosis [12]. Additionally, similar studies have confirmed that CMA is considered the first-line detection method for prenatal diagnosis [5, 13]. Our study found that CMA can detect 3.73% more chromosome abnormalities than karyotype analysis, which is consistent with previous findings [13]. However, several disadvantages of CMA have been pointed out. For example, a previous study demonstrated that CMA cannot detect low levels of mosaicism, leading to missed diagnoses [14]. Other studies have also pointed out that balanced chromosome rearrangement cannot be detected using CMA [15, 16]. On the other side, some chromosomal abnormalities would be missed if CMA was used alone. Briefly, a case diagnosed as Coffin-Lowry syndrome (disruption of CHD7) has normal CMA results but an abnormal karyotype result [17]. In our study, we also found that 12 cases had abnormal karyotype analysis, despite normal CMA results. Therefore, it is not appropriate for CMA to completely replace karyotyping in prenatal diagnosis, and a combination of the two methods is recommended.

Regarding chromosome mosaicism, FISH or CNVplex analysis was used to further evaluate the results of chromosome abnormalities in the current study. For instance, the karyotype result of case 1 was modified to 45, X, [73]/46, X, psu dic(Y) (q12) [10], based on a combination of G-banding and FISH analysis. FISH analysis, especially for the diagnosis of complex chromosome abnormalities, has set up a bridge between chromosome banding technology and molecular genetics [18]. Additionally, previous studies have demonstrated that FISH analysis can identify low-level mosaicism [19] and mosaicism for chromosomal rearrangement undetected by molecular cytogenetics [20]. Correspondingly, the combination of G-banding and FISH provides an efficient method for prenatal and postnatal chromosomal analysis [21]. CNVplex assay, a high-throughput multiplex CNVs analysis method developed by Genesky Biotechnologies, which can not only detect the abnormal number of 24 chromosomes, but also detect the deletion or duplication of the ends of chromosomes [22]. In terms of detection accuracy, CNVplex excludes possible maternal contamination, which decreases the false negative rate, thereby greatly enhancing the reliability of the results [23]. Importantly, the cost of its approach was relatively low. In our study, the CMA and CNVplex assays suggested the mosaicism for trisomy 2, indicating the value of CNVplex for prenatal diagnosis. Combined with those findings, we believed that FISH or CNVplex can enhance the reliability of prenatal and postnatal chromosomal analysis.

## 5. Conclusion

Conventional G-banding karyotyping and CMA have their own advantages and limitations (Supplementary Table 1), and it is also inappropriate that CMA completely replace karyotyping in prenatal diagnosis. Combined karyotype analysis and CMA for prenatal diagnosis can increase the reliability of detection. Additionally, FISH or CNVplex analysis was recommended to further evaluate the results when chromosomal mosaicism was detected. Those findings provided a better understanding of the clinically significant genetic variants.

## Figures and Tables

**Table 1:** Diagnostic yields in 3710 prenatal samples by karyotype analysis and CMA.

Sample type	Sample numbers	Karyotype abnormalities	CMA abnormalities	Karyotype + CMA abnormalities
Amniotic fluid	3710	201 (5.41%)	340 (9.14%)	352 (9.49%)

**Table 2:** Characteristics of prenatal samples with an abnormal karyotype and a normal CMA.

Cases	Characteristics	Karyotype results	CMA results
1	Low number of noninvasive chromosomes	45, X, [73]/46, X, psu dic (Y) (q12) [10]	46, XY
2	Noninvasive DNA suggested sex chromosome anomalies (47, XXY)	47, XXY [2]/46, XY[48]	46, XY
3	Noninvasive suggested X chromosome abnormalities	45, X [2]/46, XX[48]	46, XX
4	Fetal intrauterine growth restriction	47, XX, +20 [3]/46, XX[97]	46, XX
5	High risk of trisomy 21	45, X [8]/46, XX[42]	46, XX
6	Low F-*β*HCG (MOM)	47, XYY [5]/46, XY[45]	46, XX
7	High number of noninvasive chromosomes	46, XY, *t*(1; 9) (p31; q31)dn	46, XY
8	Bilateral nasal bone dysplasia in fetus	46, XY, *t*(4; 8) (q21; q24.1)dn	46, XY
9	High F-*β*HCG (MOM), risk value of trisomy 21 was 1/237	46, XY, *t*(12; 13) (p11.23; q12)dn	46, XY
10	NT: 2.82 mm, reversed a-wave in the fetal ductus venosus	46, XX, ins(2; 16) (p13; q12q24), *t*(2; 5) (q23; p15.1), *t*(2; 8) (p13; p22)dn	46, XX
11	Risk value of trisomy 21 was 1/256	46, XY, *t*(2; 10; 14) (q23; q22; q32)dn	46, XY
12	Nasal bone was invisible, right choroid plexus cyst	46, XX, *t*(4; 15) (p14; q11.1)dn	46, XX

**Table 3:** Characteristics of prenatal samples with a normal karyotype and an abnormal CMA.

Cases	Characteristics	Karyotype results	CMA results
13	Single umbilical artery, strong echo of left heart, fetal intrauterine growth restriction	46, XX	Mosaicism for trisomy 2
2	Noninvasive DNA suggested high risk of trisomy 15	46, XY	Mosaicism for trisomy 15
3	Short long bones, separation of left kidney and renal pelvis, dilation of ureter, gallbladder not shown, trisomy 2	46, XX	Mosaicism for trisomy 2
4	≥35 years old, NT > 95th	46, XX	Mosaicism for trisomy 14

## Data Availability

All the data generated during this study are included within this article.

## Abbreviations

CMA: Chromosome microarray analysis
FISH: Fluorescence in situ hybridization
CNVs: Copy number variants
MCC: Maternal cell contamination
ACOG: American College of Obstetrics and Gynecology.

## Supplementary Materials

Supplementary Materials Supplementary Table 1 shows the advantages and limitations of conventional G-banding karyotyping and CMA.
