# Supplementary material for: Assessment of Combined Karyotype Analysis and Chromosome Microarray Analysis in Prenatal Diagnosis: A Cohort Study of 3710 Pregnancies
Source: Genet Res (Camb). 2022 Dec 29;2022:6791439. doi: 10.1155/2022/6791439 (PMC9815932; doi:10.1155/2022/6791439)
Supplement: Supplementary Materials — Supplementary Table 1 shows the advantages and limitations of conventional G-banding karyotyping and CMA. [file 6791439.f1.docx]

**Supplementary Table 1** The comparison of advantages and limitations of G-band karyotyping and Chromosome microarray

| Approach | Advantages | Limitations |
| --- | --- | --- |
| G-band karyotyping | 1) Clear chromosome bands;  2) Can be distinguished under ordinary microscope;  3) The specimen can be stored for a long time; | 1) Long detection period;  2) Low resolution; |
| Chromosome microarray | 1) Identify small microdeletions and duplications  2) Helpful to understand the disease related copy number variations  3) Deemed as first-line test in pregnancies with a priori low risk | 1) Cannot detect low levels of mosaicism;  2) Cannot detect balanced chromosome rearrangement |
